# Is Demography Destiny? Application of Machine Learning Techniques to Accurately Predict Population Health Outcomes from a Minimal Demographic Dataset

**DOI:** 10.1371/journal.pone.0125602

**Published:** 2015-05-04

**Authors:** Wei Luo, Thin Nguyen, Melanie Nichols, Truyen Tran, Santu Rana, Sunil Gupta, Dinh Phung, Svetha Venkatesh, Steve Allender

**Affiliations:** 1 Centre for Pattern Recognition and Data Analytics, School of Information Technology, Deakin University, Geelong, Victoria, Australia; 2 World Health Organization Collaborating Centre for Obesity Prevention, Deakin University, Geelong, Victoria, Australia; City University of New York (CUNY), UNITED STATES

## Abstract

For years, we have relied on population surveys to keep track of regional public health statistics, including the prevalence of non-communicable diseases. Because of the cost and limitations of such surveys, we often do not have the up-to-date data on health outcomes of a region. In this paper, we examined the feasibility of inferring regional health outcomes from socio-demographic data that are widely available and timely updated through national censuses and community surveys. Using data for 50 American states (excluding Washington DC) from 2007 to 2012, we constructed a machine-learning model to predict the prevalence of six non-communicable disease (NCD) outcomes (four NCDs and two major clinical risk factors), based on population socio-demographic characteristics from the American Community Survey. We found that regional prevalence estimates for non-communicable diseases can be reasonably predicted. The predictions were highly correlated with the observed data, in both the states included in the derivation model (median correlation 0.88) and those excluded from the development for use as a completely separated validation sample (median correlation 0.85), demonstrating that the model had sufficient external validity to make good predictions, based on demographics alone, for areas not included in the model development. This highlights both the utility of this sophisticated approach to model development, and the vital importance of simple socio-demographic characteristics as both indicators and determinants of chronic disease.

## Introduction

It is well known in public health that socio-demographic factors are key determinants of health and wellbeing in a population [1,2]. Much research exploring determinants of health collects these measures routinely but largely ignores these factors, beyond simply adjusting or stratifying statistical analyses. Demographic factors are also usually very difficult, or in some cases impossible, to change and so little direct attention is paid to their relative contribution to health or disease and the contribution itself is rarely quantified. While standard demographics are included in most studies and used within standard analytic approaches these factors may be underestimated and the influence of inequalities between socio-demographic groups overlooked. Socio-demographic factors are known to be important, yet the full extent of their contribution to health and illness is not necessarily obvious.

New machine learning techniques provide a potentially very powerful means of building high quality predictive models, particularly in situations where relationships between variables are complex and may not be fully elucidated using standard statistical techniques. In recent years, a variety of machine learning approaches have been applied to health issues and have demonstrated high quality and valid predictions [3–6], although to date these approaches have focused predominantly on individual-level clinical records and outcomes. Machine learning techniques may be particularly useful in exploring health issues where complex interactions exist between a large number of determinants and the outcomes of interest, in ways that traditional regression approaches may not be able to adequately model, or to identify *a priori*. There are a number of ways that the proposed machine learning models may contribute to addressing challenges in public health. These new techniques may be able to provide insight not only into existing relationships but also provide higher predictive utility than traditional approaches, expanding the role of socio-demographic data from merely contributing to explanations of patterns of health, to predicting their distribution in communities. A major challenge in monitoring population health is the regularity, timing and granularity of data available. If they are able to achieve sufficient precision, modelled estimates can play an important part in strategic decision making[7], and machine learning models may provide a route to this required level of precision. Such predicted statistics, based only on a small set of population-level characteristics, may be able to fill gaps in data collection from more traditional sources, or facilitate development of estimates for smaller geographic regions, for which it may not be feasible or cost-effective to produce survey-based estimates.

Another persistent challenge in population health is identifying the characteristics of communities, environments and policies that promote health and wellbeing. These characteristics have the potential to inform both intervention design and future community planning to support health. There is potential that analysis of situations where the measured rates of health outcomes diverge substantially from demographic-based predictions may help to identify such areas of best practice and provide much needed information, in the style of ‘natural experiments’ evaluations, to inform future health promotion policies and activities[8]. Finally, it is often extremely difficult to compare the health outcomes of states or other areas where the population demographic profiles may be very different. It may be difficult, for instance, for policy-makers to accurately compare states with much younger or older than average population profiles, to determine whether the health system is performing well. Divergence of observed from predicted values may provide a method for ranking states’ performance in promoting population health that takes account of the full and extensive effect of demographic characteristics [8]. States or countries may then be ranked, or league tables generated according to the measured rates of disease outcomes compared to rates predicted by demographic variables alone. This provides a fairer method of comparing achievements in prevention and treatment across states with very diverse population demographics.

In this paper, we explore the extent to which a simple set of socio-demographic factors can be used to predict the prevalence of non-communicable diseases and risk factors at the state level in the United States. Further, we explore the extent to which ‘demography is destiny’ in the observed health outcomes, or whether there is evidence that some states have achieved significantly better or worse outcomes than we would expect based on their demography alone.

## Methods

Using data for 50 American states (excluding Washington DC) from 2007 to 2012, we constructed a machine learning model to predict the prevalence of six non-communicable disease (NCD) outcomes (four NCDs and two major clinical risk factors), based on population socio-demographic characteristics from the American Community Survey[9]. The 50 states were randomly sampled into two groups: a derivation group of 30 states and a validation group of 20 states. The outcome data from the derivation group of states was used to ‘train’ the system and develop the model, and was therefore assumed to have all data about NCDs and risk factors. Data from the validation group was not used in the model development, but was used to test the accuracy of the model’s predictions, emulating regions with no existing NCD surveillance program.

**Fig 1:**
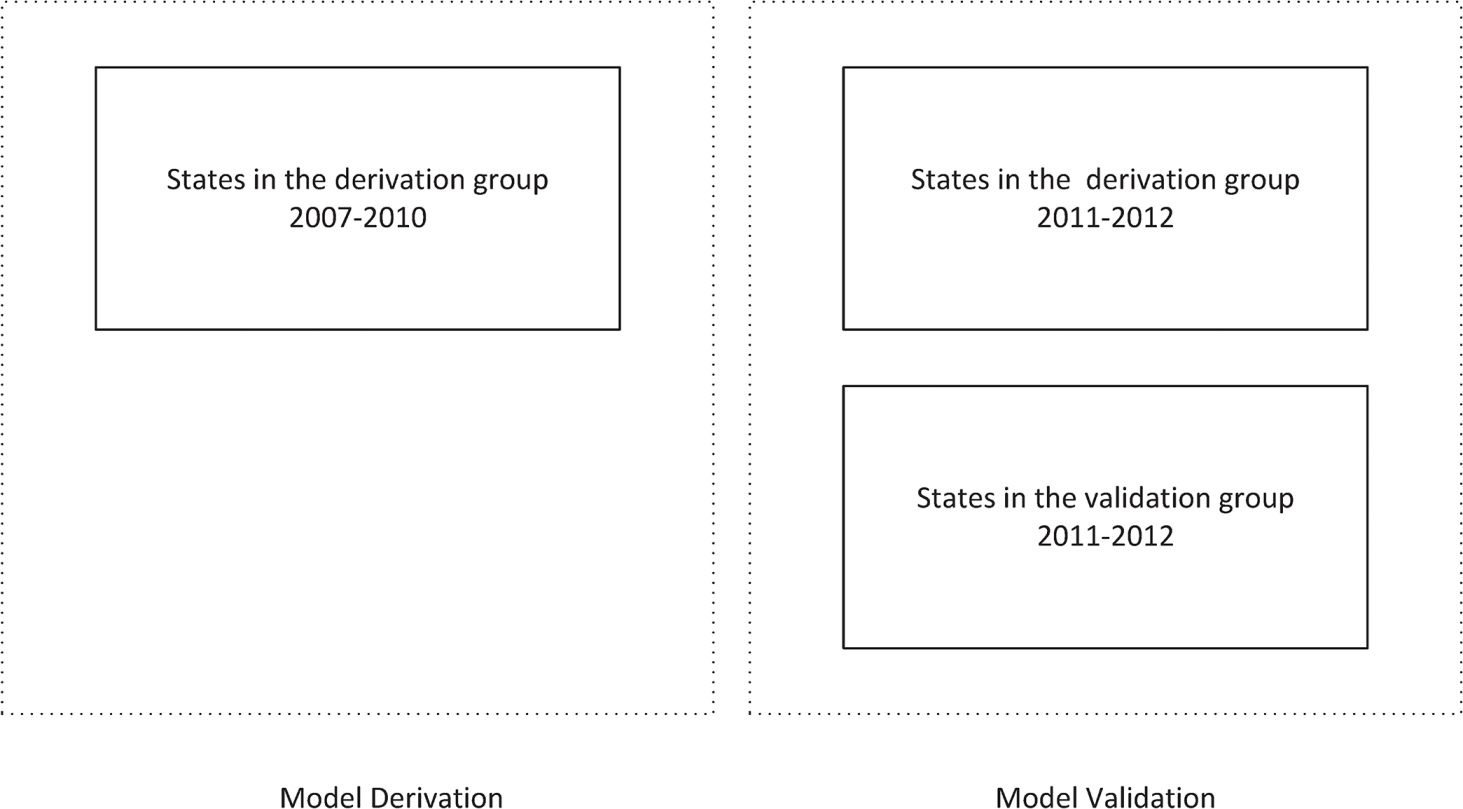
Data used for model derivation and validation. The 50 states were randomly sampled into a derivation group of 30 states and a validation group of 20 states (See Fig 2). Models were derived from data of the derivation group during years 2007 to 2010, and were validated using 2011–2012 data of both the derivation group and the validation group.

**Fig 2:**
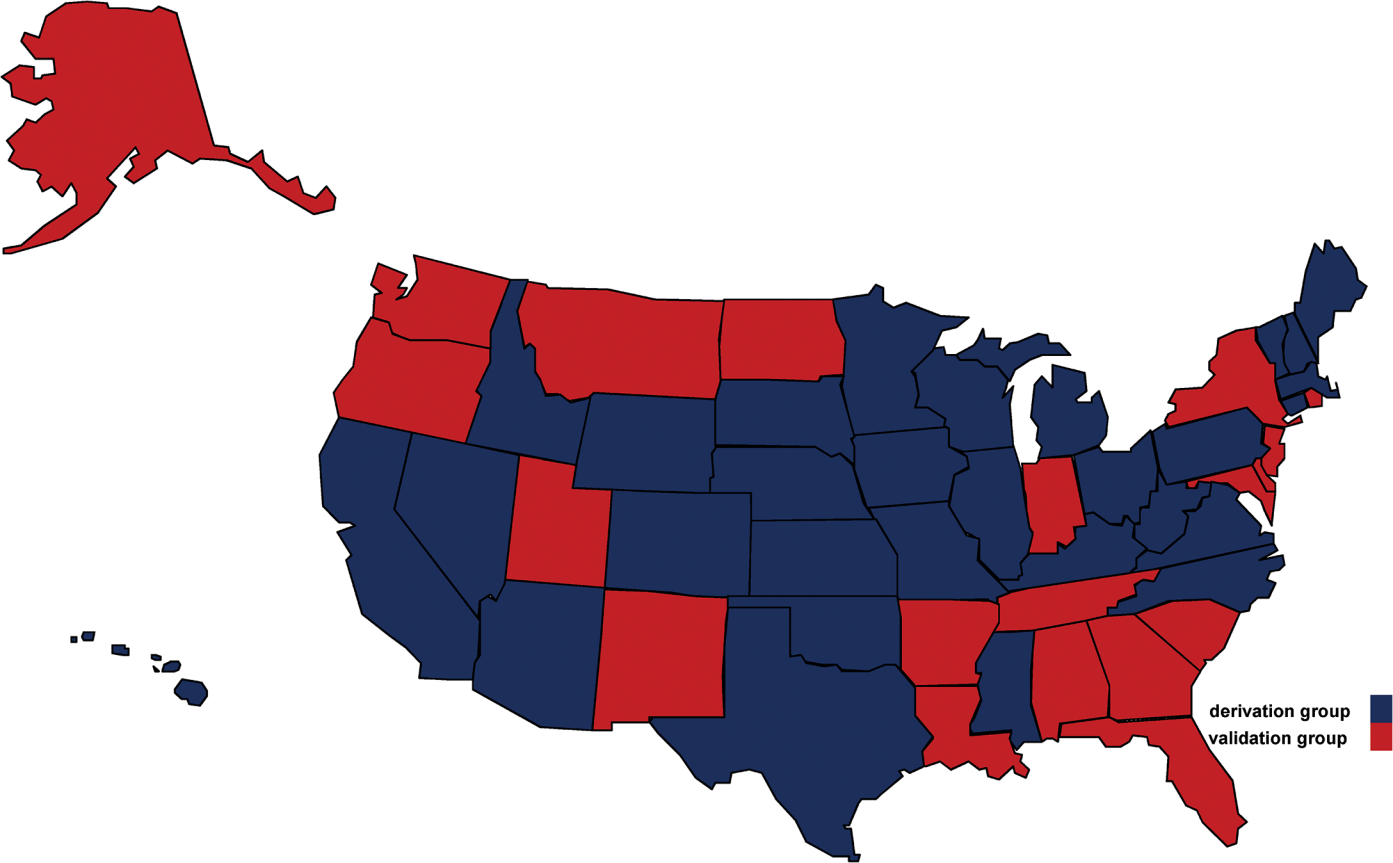
States allocated to the derivation (training) group and the validation (testing) group for model development. 30 states from the derivation group were coloured blue; 20 states from the validation group were coloured red.

For each of the six outcomes, a model predicting the prevalence was derived using the derivation group data during 2007–2010. The model was then validated using both the chronically separated data (for 2011–2012) from the derivation group and the out-of-sample data from the validation group (See Figs 1 and 2). The feasibility of using the model across states was measured through both the model's predictive performance on the validation group and the performance difference between the (chronically separated) data from the derivation group and the validation group.

### Dependant variables: NCD prevalence data from the Behavioral Risk Factor Surveillance System

All data for state-level prevalence of NCDs and risk factors was drawn from the Behavioral Risk Factor Surveillance System (BRFSS) [10,11]. The BRFSS is an ongoing telephone-based population health survey, conducted by the United States Centers for Disease Control and Prevention (CDC). Survey results, including state-based population estimates of diseases and risk factors, are published online annually by the CDC. At the time of writing, the most recent data available were for year 2012. Table 1 shows the risk factors included in the study[10]. The risk factor “High blood pressure” was only available in the dataset every second year (2007, 2009, and 2011). All other variables have annual prevalence data.

**Table 1:** Independent and dependent variables included in the study, measuring proportions of population for different survey responses.

Variable	Details and categories
Independent variables (American Community Survey)	
Age	Proportion (%) of population aged: 18–25 years* / 25–34 / 35–44 / 45–54 / 55–64 / 65 years and over
Sex	Proportion (%) of males in population
Race	Proportion (%) of population: White* / Black or African American / Asian / Native Hawaiian or Other Pacific Islander / American Indian or Alaska Native / Two or more races / Other
Household income	Proportion (%) of population with annual total household income: <USD $15,000* / $15,000-$24,999 / $25,000-$34,999 / $35,000-$49,999 / $50,000 or more
Employment status	Proportion (%) of population: Employed* / unemployed
Marital status	Proportion (%) of population: Married* / Divorced / Widowed/ Separated / Never married
Education	Proportion (%) of population with the following levels of formal education: Did not complete high school* / high school graduate / some college or associated degrees / Bachelor degree or higher
Dependent variables (Behavioral Risk Factor Surveillance Survey)	
High blood pressure	State-level proportion of adults (%) who have ever been told they have high blood pressure. Self-reported.
Obese	State-level prevalence (%) of population with body mass index (BMI) greater than 30kg/m^2^. Based on self-reported weight and height.
Cardiovascular Disease—Angina or coronary heart disease	State-level prevalence (%) of self-reported chronic heart condition. “Has a doctor, nurse, or other health professional ever told you that you had angina or coronary heart disease?”
Cardiovascular Disease—Heart attack	State-level prevalence (%) of self-reported history of AMI. “Has a doctor, nurse, or other health professional ever told you that you had a heart attack, also called a myocardial infarction?”
Cardiovascular Disease—Stroke	State-level prevalence (%) of self-reported history of stroke. “Has a doctor, nurse, or other health professional ever told you ever told you that you had a stroke?”
Diabetes	State-level prevalence (%) of lifetime diabetes diagnosis. Excluding gestational diabetes only diagnoses and borderline / pre-diabetes. “Has a doctor, nurse, or other health professional ever told you that you have diabetes?”

*reference category.

### Independent variables: Socio-demographic data from the American Community Survey

Data on demographic and social characteristics of each state were extracted from the American Community Survey (ACS) [9]. The ACS collects a wide range of community characteristics through mail and telephone calls. Data aggregated at the state level are updated annually. In this study, state-level summary data were included for the following population socio-demographic variables: age, gender, race, household income, employment status, marital status and education. The data used in the model development were continuous variables representing the percentage of the state population falling within each category for each characteristic. The details of the variables and the categories for each of the characteristics are shown in Table 1.

Some demographic data can also be found from the BRFSS, which covers veteran status, marital status, number of children in household, education level, employment status, and income level (See Section 8.5 to 8.10 of the BRFSS codebook). We chose not to use these demographic variables from BRFSS, to reduce potential selection bias shared by the independent variables and the dependent variables. By using independent variables from an independent source, we should have generalizable results. In particular, for a region with no BRFSS-like survey system in place (e.g., many low-to-median income nations), our model can still predict the prevalence of particular health outcomes, as long as some basic demographics data are available.

In the same consideration, we chose not to include prior-year health outcomes (dependent variables) as independent variables. Although this may increase the accuracy of prediction, it relies on the presence of a BRFSS-like system.

### Derivation and validation of models for inferring NCD prevalence from census data

For each NCD or risk factor prevalence (denoted *Y*), a regression model was derived to estimate the prevalence of the risk factor in a region:

Y=c+β⋅(ACS variables) (1)

Regression models in the form of (1) were fitted using the data from the 30 states from the derivation group during 2007 to 2010 (See Fig 1). Each observation unit corresponds to a combination of some state and some year (e.g., Arizona and 2007, Arizona and 2008, and so on). Due to the large number of variables involved, the regression model was fitted using least square with lasso penalization [12] on the regression coefficients ***β***. Lasso has a control parameter that decides the number of variables being selected for a given health index, and this was determined by 10-fold cross-validation. Apart from the lasso regression, a number of other general machine learning methods—regression with stepwise feature selection, group lasso, random forest, and Gaussian process regression—were also used to fit the data, ensuring the results were robust against the choice of learning methods. The estimated models were validated using out-of-sample data from 1) 2011–2012 data from the derivation group, and 2) 2011–2012 data from the validation group. In validation, the model generated the estimates for NCD and risk factors based on the ACS variables from the validation sample. The estimates were compared with the prevalence reported by BRFSS. Correlation between the predicted and observed variables was calculated. In addition, states were classified into prevalence quintiles for each of the six NCD outcomes, according to both observed and predicted values, and the quintile classifications compared for each outcome and year.

While the results for the validation group directly measure the model’s prediction performance, it is the performance difference between the derivation cohort and the validation cohort that can be used to assess the degree of over-fitting in the model and its generalizability to unseen data.

## Results

The 2011 mean prevalence of the six outcomes, as per the BRFSS, was similar between the states in the derivation and validation groups (Table 2). The two risk factors—high blood pressure and obesity—were the most prevalent, affecting between one in four and one in three of those surveyed. The prevalence of each cardiovascular disease was less than 5% of the population in both derivation and validation states.

**Table 2:** Prevalence of NCD and risk factors for the derivation group states and the validation group states at year 2011, defined by proportion of positive responses to the corresponding BRFSS questions.

	State Prevalence, mean (sd)	
	Derivation group	Validation group
**Intermediate risk factor**		
High blood pressure	31.1 (3.3)	32.5 (4.1)
Obese	27.6 (3.2)	27.8 (2.7)
**Diseases**		
Cardiovascular Disease-Angina or CHD	4.2 (0.9)	4.3 (0.8)
Cardiovascular Disease-Heart attack	4.4 (0.9)	4.5 (0.9)
Cardiovascular Disease-Stroke	2.9 (0.5)	3.1 (0.7)
Diabetes	9.4 (1.3)	9.7 (1.4)

**Fig 3:**
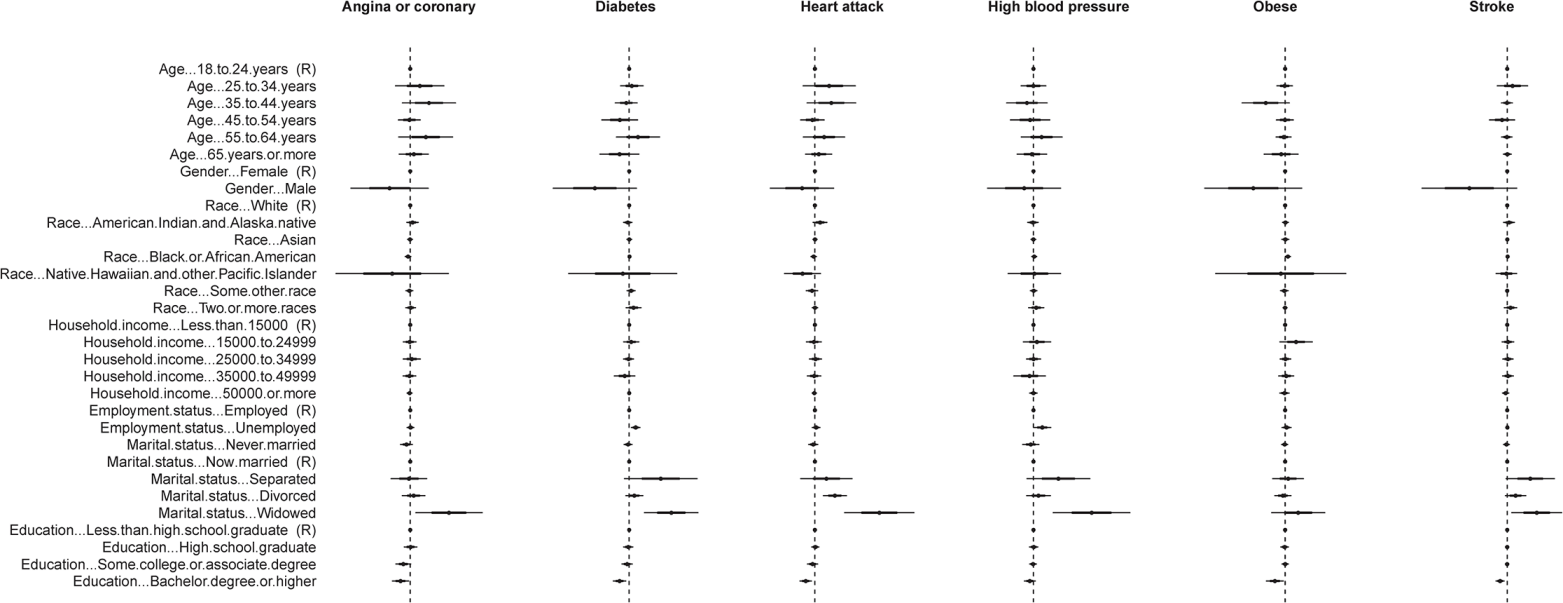
Coefficients of independent demographical variables in models predicting different NCD and risk factors. The dashed lines show the zeros. The center dots show the means of coefficients. The thicker bars show ± *sd* and the thinner bars show ± 2*sd* based on 1000 models with resampling of the training data.

Standardised coefficients, showing the contributions of the independent socio-demographic variables to the models are shown in Fig 3. The strongest independent predictor of state-level health outcomes was education levels, in particular the proportion of the state’s population with a bachelor’s degree or higher.

**Fig 4:**
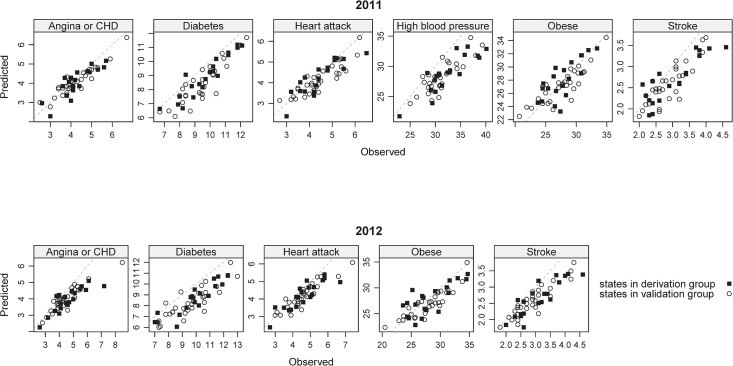
Predicted vs Observed values of state NCD and risk factor prevalence. Agreement between the predicted and the observed values is reflected by proximity of the points with the diagonal dashed lines.

The performance of the prediction models, using only information about the states’ socio-demographic profiles, compared with the out-of-sample observed data is shown in Table 3, and as scatter plots in Fig 4. Pearson correlations between the predicted and observed values ranged between 0.823 (prevalence of high blood pressure) and 0.906 (Angina or CHD) for the 2011 and 2012 estimates of the derivation states. Correlations in the validation states were lower for the same years, varying between 0.753 (prevalence of obesity) and 0.911 (prevalence of diabetes). The median relative absolute error ranged between 0.184 (prevalence of stroke) and 0.052 (prevalence of obesity). Similar numbers result from other statistical models (see Table 4). In general, the model appeared to slightly under-estimate the absolute prevalence of the outcomes in 2011 and somewhat more so in 2012, reflecting in negative bias in the validation data set. When the values were used to classify states into quintiles of NCD prevalence, the model predictions performed well, and the performance was similar in both years of validation data. Overall, 50% of the 550 state-year-NCD prevalence rate combinations were classified into the same quintile by the predictions of the demographic-based machine learning model as by their survey-obtained observed data, while 92% of estimates were classified within one quintile (plus or minus) of the observed quintile. There was no difference between 2011 and 2012 in the proportion of states correctly classified into quintiles of NCD prevalence (data not shown).

**Table 3:** Accuracy of risk factor and disease estimates evaluated using out-of-sample data from 2011 and 2012.

	RMSE* (bias)		Median Absolute Error (Median Relative Absolute Error)		Pearson correlation (95% CI)	
	Derivation	Validation	Derivation	Validation	Derivation	Validation
**Intermediate risk factor**						
High blood pressure	3.436 (-2.901)	4.159 (-3.601)	2.672 (0.097)	3.491 (0.12)	0.823 (0.658–0.913)	0.864 (0.683–0.945)
Obese	1.723 (-0.7)	2.084 (-0.089)	1.196 (0.043)	1.515 (0.052)	0.872 (0.794–0.922)	0.753 (0.577–0.862)
**Diseases**						
Angina or CHD	0.528 (-0.299)	0.72 (-0.457)	0.298 (0.076)	0.458 (0.131)	0.906 (0.847–0.943)	0.806 (0.698–0.907)
Heart attack	0.541 (-0.361)	0.552 (-0.304)	0.398 (0.097)	0.385 (0.103)	0.9 (0.837–0.939)	0.861 (0.751–0.924)
Stroke	0.409 (-0.301)	0.537 (-0.399)	0.242 (0.091)	0.463 (0.184)	0.876 (0.794–0.922)	0.866 (0.759–0.927)
Diabetes	1.381 (-1.158)	1.188 (-1.008)	1.115 (0.126)	0.908 (0.103)	0.851 (0.762–0.909)	0.911 (0.838–0.952)

*RMSE stands for Root Mean Squared.

**Table 4:** Prediction performance of different models.

	RMSE on validation group (bias)			
	Stepwise regression	Group lasso	Random forest	Gaussian process regression
**Intermediate risk factor**				
High blood pressure	2.324 (-1.231)	3.35 (-2.652)	4.387 (-3.676)	3.141 (-2.438)
Obese	2.194 (-0.658)	2.059 (-0.451)	2.512 (-0.765)	2.113 (-0.852)
**Diseases**				
Cardiovascular Disease - Angina or CHD	0.818 (-0.467)	0.657 (-0.376)	0.678 (-0.308)	0.489 (-0.121)
Cardiovascular Disease - Heart attack	0.595 (-0.164)	0.518 (-0.24)	0.64 (-0.328)	0.547 (-0.288)
Cardiovascular Disease - Stroke	0.513 (-0.312)	0.491 (-0.327)	0.493 (-0.36)	0.57 (-0.343)
Diabetes	1.091 (-0.839)	1.166 (-0.999)	1.498 (-1.204)	1.432 (-1.05)

*The following software packages were used: the stepAIC function from the MASS R package [13](for stepwise regression), the glmnet R package [14] (for group lass), the randomForest R package [15] (for random forest), and the GPML Matlab toolbox [16] (for Gaussian process regression). For Gaussian process regression, feature selection was first performed with Hilbert-Schmidt Independence Criterion Lasso [17]. The mean function was a constant function of the mean prevalence in the training set. The covariance function was the squared exponential with a maximum allowable covariance 10 and a length parameter 10.

In the exploration of states which had actual (observed) prevalence rates that were substantially different from the rates predicted by the demographic model (Table 4), many more states had observed values that were more than 10% greater (worse) than expected than had observed values lower than expected. Almost all states (47) had observed values at least 10% worse than predicted for at least one indicator in at least one of the validation years. This is consistent with the observation that the model generally underestimated prevalence of NCDs in the later years of data. There were just 15 states which had at least one NCD prevalence outcome in 2011 or 2012 that was lower (better) than the predicted prevalence by at least 10%. In 2011, 5 states (Alaska, Colorado, Iowa, New Mexico and Rhode Island) were better than expected on at least two indicators. In 2012, Montana was the only state in which at least two indicators were 10% better than predicted.

## Discussion

This study found that regional prevalence estimates for non-communicable diseases can be reasonably predicted (generally correlated with observed data at > 0.80), using a very simple set of routinely collected socio-demographic characteristics of the population, with the application of machine learning models to existing datasets. This highlights both the utility of this sophisticated approach to model development, and the vital importance of simple socio-demographic characteristics as both indicators and determinants of chronic disease.

The derived model’s predictions of NCD prevalence, across 6 outcomes, for 2 years of data (that were not included in the original model development), were highly correlated with the observed data, in both the states included in the derivation model (median correlation 0.88) and those excluded from the development for use as a completely separated validation sample (median correlation 0.85), demonstrating that the model had sufficient external validity to make good predictions, based on demographics alone, for areas not included in the model development.

While the results are promising, there are some potential weaknesses inherent in the nature of the data used. Firstly, there are weaknesses in the data available for NCD prevalence estimates from the BRFSS, as they rely on somewhat subjective, self-reported indicators of chronic disease[10]. Potential issues with these indicators include recall and reporting biases, and biases in relation to systematic differences in diagnosis levels of disease. As a result, these indicators do not necessarily provide a ‘gold standard’ estimate of prevalence either for training or testing the machine learning models, and may explain some of the variation in the precision of estimates.

The demographic data in this study was obtained from the American Community Survey (ACS). The complex design of ACS and BRFSS implies that important differences exist between these two surveys. First, the sampling units in ACS are Household Units (or Group Quarters), while the sampling units in BRFSS are owners of a telephone number. Next, ACS relies on mail, internet, telephone, and in-person visits to maximize the response rate, while BRFSS relies on telephone (both landline and mobile phones) interviews only. Finally, BRFSS data are collected by each state health department. Although uniform guidelines exist, each state may carry out the survey differently. Despite these and other differences, both ACS and BRFSS aim at the same target population (the state population of age 18 and above), with sophisticated weighting methods. By using two separate data sources for demography and health outcomes, it is likely that any shared selection bias would be minimised, although the reported model accuracy may look lower.

BRFSS was based on landline numbers until year 2011, when mobile numbers were included as well. At the same time, sampling weights were also changed. Such changes in survey methodology may also decrease the prediction accuracy reported here.

Both ACS and BRFSS data were prevalence proportion estimates with survey weighting accounted for. Any interpretation of the results should be made with that in mind.

A further possible limitation, particularly in relation to applying the models for forward predictions of NCD trends, is the inability of the model to adapt to secular changes in NCD prevalence, which are likely to occur at a much faster rate than changes in the demographic profile of the regions. This provides an explanation of why all of the models investigated in this study consistently underestimated prevalence for later years (Table 4), in particular for 2012 (Table 5), while the relative ranking of states remained just as accurate. This under-estimation may be more pronounced for models based on the lasso penalty, which shrink model coefficients towards the zero. A key strength of the study on the other hand, was that the demographic data used are simple, widely available, and for this study were drawn from an entirely separate data collection system.

**Table 5:** Number of states with observed values differing from predicted values by greater than 10% of estimate, by outcome and year.

	NCD prevalence >10% better than predicted by demographic model		NCD prevalence >10% worse than predicted by demographic model	
	2011	2012	2011	2012
CVD—Angina/CHD	6	0	9	23
CVD—AMI	5	2	17	24
CVD—Stroke	4	1	21	27
Diabetes	1	2	15	26
High blood pressure	0	n/a	24	n/a
Obesity	2	4	3	5

Predicting outcomes based on socio-demographic (e.g. census) data has several potential future uses beyond simply quantifying the central role that demographics characteristics play in determining population health outcomes. The level of prediction accuracy achieved in this demonstration, could be applied to fill gaps in data collection from more traditional sources.

The approach taken in this paper is related to, although distinct from, the practice of ecological inference, and some cautions must therefore be applied to interpretation. Ecological inference typically uses aggregate data to infer conclusions about individual relationships[18]. The technique demonstrated in this paper used aggregate (state level) demographic characteristics to predict state level NCD prevalence. The results do not, however, provide any insight into individual-level associations between demographic characteristics and NCDs. That is, while the model uses state demographic profiles to accurately predict state-level NCD prevalence, it does not and cannot infer that an *individual* with certain characteristics would have a higher or lower likelihood of suffering from an NCD. To avoid potentially misleading conclusions and an ‘ecological fallacy’ the model’s predictions must remain at and be interpreted at an area level. Further, it is also important that the results of the machine predictions are interpreted with caution, and that any extrapolation or future predictions are not stretched too far from the ‘training’ data. It is plausible that the observed ecological associations may not be stable over time. If models trained using aggregate data estimated at one level (e.g. state) are to be applied to predict prevalence at a different geographic level (e.g. regions), care must be taken to validate the approach with observed data where possible, as relationships between population demographics and NCD prevalence may differ at different geographical levels[19].

When richer data are available, more accurate prevalence estimates may be achieved. In the well-known Global Burden of Disease Study 2010 [20], a carefully crafted model produces prevalence estimates from 179 covariates. In particular, random effects for different levels of geographic regions model the prevalence similarity of neighbouring regions; data from previous years were included to improve accuracy. In this article, we focus on the utility of widely available socio-demographic survey data, and aim for a model that generalizes to a region with no previous data. Hence we opted for a simpler model with only demographic variables. Such a model does not assume data from multi-level geographic partitions or from previous years.

Analysis of situations where the measured rates of health outcomes diverge substantially from machine predictions may help to identify areas of best practice or areas with greater need for investment in action and policy to prevent and manage the disease. In this study, a small number of states were found to have NCD prevalence levels substantially better than would be expected based on their demographic profiles. It can be deduced that those states outperforming their predicted values by the greatest amount have endogenous characteristics, environments, or most importantly, policy settings, which promote health and may be protective against NCDs. These findings open up possibilities for future comparative analysis to identify the most important modifiable influences, which may hold promise for intervention and policy development.

For service planning purposes, this study powerfully illustrates the strength to which simple demographic characteristics can be used to predict likely disease burden, with the application of sophisticated modelling techniques. When considering these results from a public health intervention perspective, they are a striking reminder of the crucial importance to population health of so many variables that we routinely diminish or 'adjust for', when pursuing further evidence about our modifiable risk factors of interest.

The research described here is a simple demonstration of the potential for machine learning techniques to contribute to the field of public health research, as well as a clear reminder of the central importance of underlying socio-demographic factors in determining health. The technique demonstrated raises the possibility of future low cost approaches to appropriately estimating disease burden in regions where data collection is infrequent or difficult. There are numerous questions still to be answered, however, and application of the technique in a variety of contexts is needed to determine the extent to which results can be extrapolated to different environments or disease profiles. It remains to be determined, for example, whether the models can perform as well in situations where outcomes or exposures (demographics) are more heterogeneous between regions, or less stable over time.

### Conclusions

Demography appears to play a very important role in population health ‘destiny’ in terms of important health outcomes. The findings of our research, using socio-demographic characteristics to model and accurately predict chronic disease implies both that fundamental population characteristics underpinning patterns in health and illness deserve close attention, and that sophisticated analysis of these characteristics can provide useful insights for understanding observed trends in population health and to inform future strategic decision making for improved health outcomes.

## Supporting Information

S1 Appendix Supplementary table and figures. (DOCX)

S1 Dataset BRFSS and ACS data used in the experiment. (ZIP)
