# Supplementary material for: Is Demography Destiny? Application of Machine Learning Techniques to Accurately Predict Population Health Outcomes from a Minimal Demographic Dataset
Source: PLoS One. 2015 May 4;10(5):e0125602. doi: 10.1371/journal.pone.0125602 (PMC4418831; doi:10.1371/journal.pone.0125602)
Supplement: S1 Appendix — (DOCX) [file pone.0125602.s001.docx]

# Appendix

Table A1 States in the derivation and validation groups.

| **Derivation group** | **Validation group** |
| --- | --- |
| Arizona  California  Colorado  Connecticut  Hawaii  Idaho  Illinois  Iowa  Kansas  Kentucky  Maine  Massachusetts  Michigan  Minnesota  Mississippi  Missouri  Nebraska  Nevada  New Hampshire  North Carolina  Ohio  Oklahoma  Pennsylvania  South Dakota  Texas  Vermont  Virginia  West Virginia  Wisconsin  Wyoming | Alabama  Alaska  Arkansas  Delaware  Florida  Georgia  Indiana  Louisiana  Maryland  Montana  New Jersey  New Mexico  New York  North Dakota  Oregon  Rhode Island  South Carolina  Tennessee  Utah  Washington |


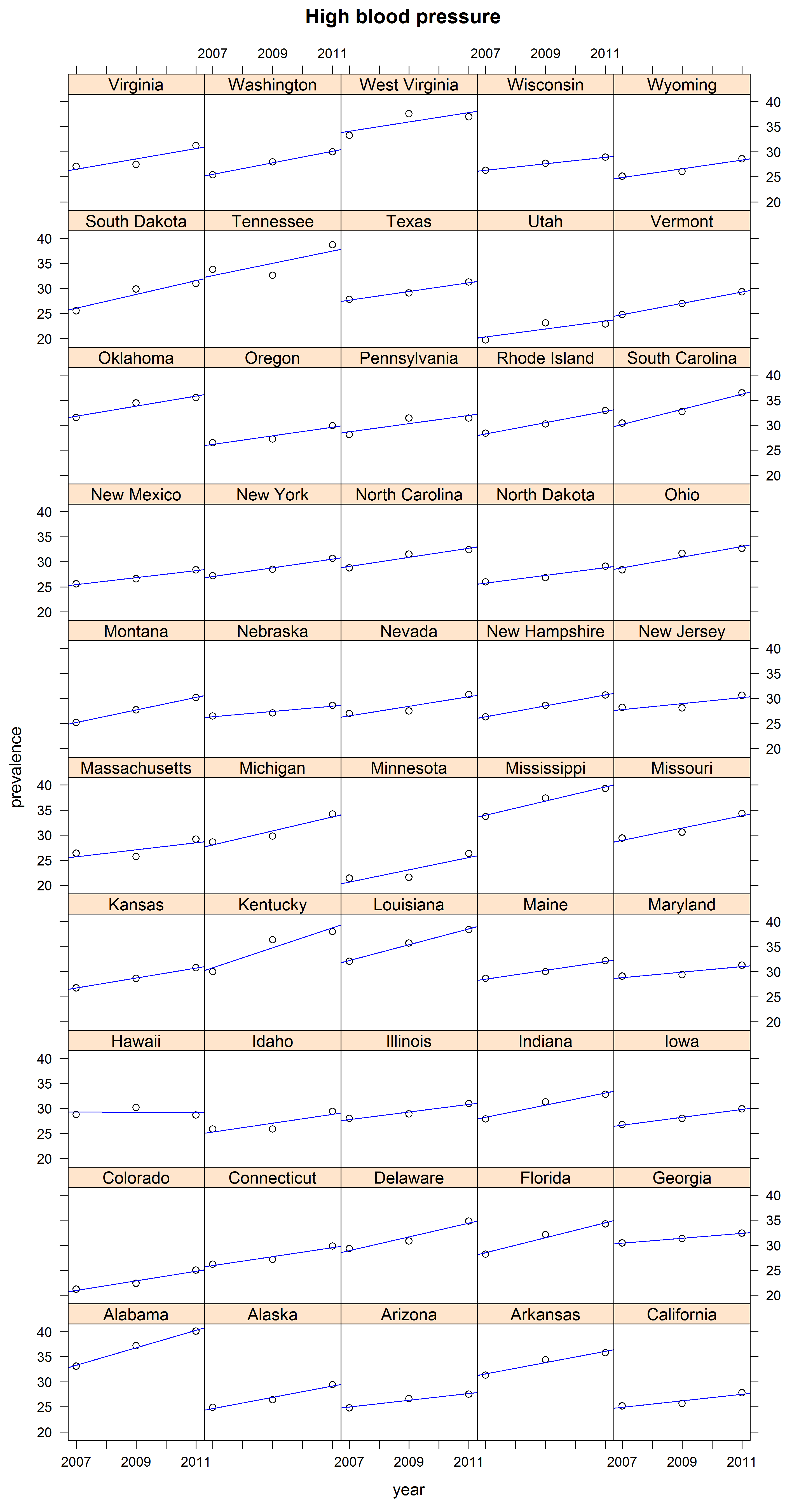


Figure A1 BRFSS reported prevalence of diseases and risk factors for year 2007-2012: High blood pressure.


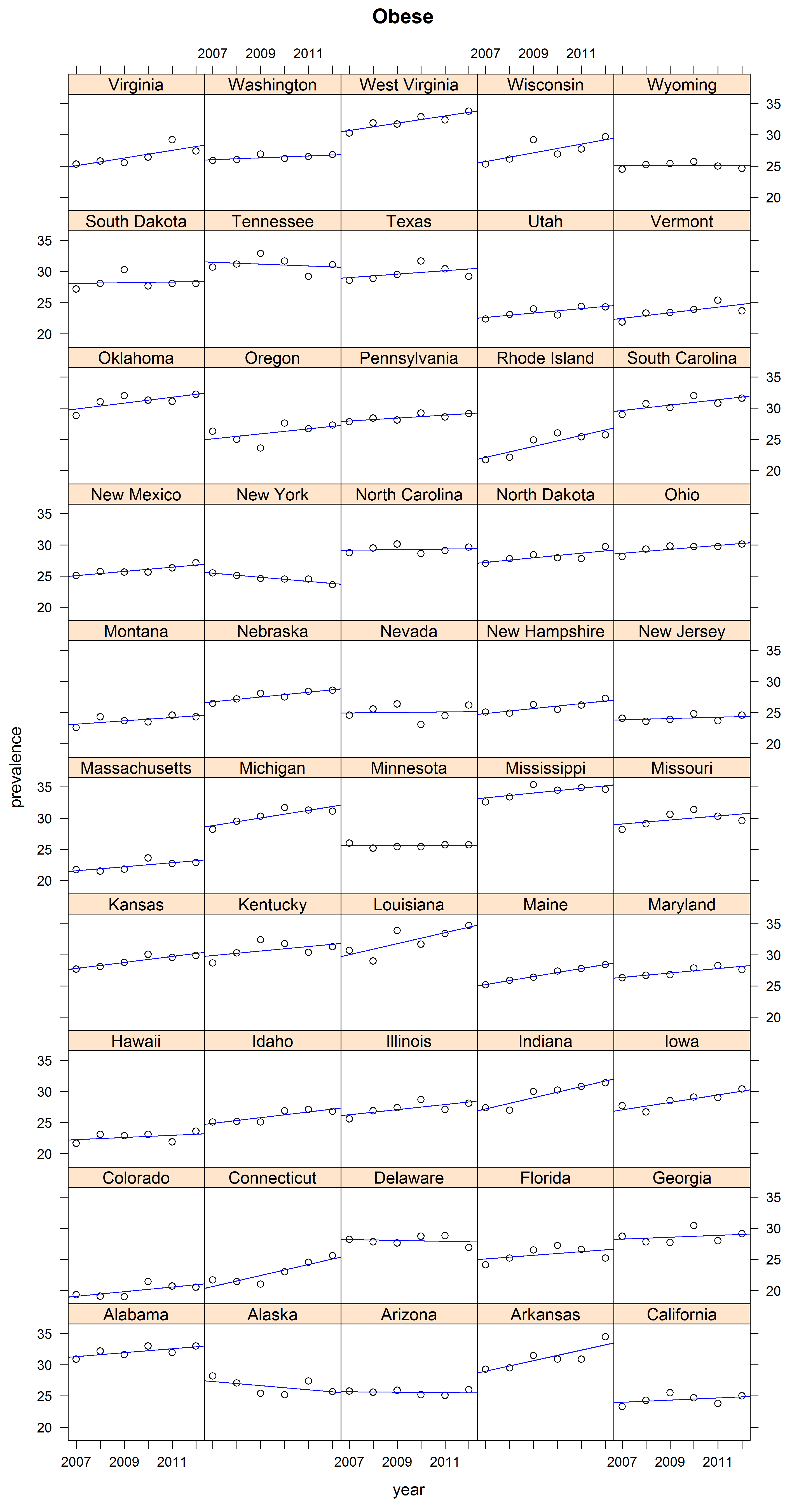


Figure A2 BRFSS reported prevalence of diseases and risk factors for year 2007-2012: Obese.


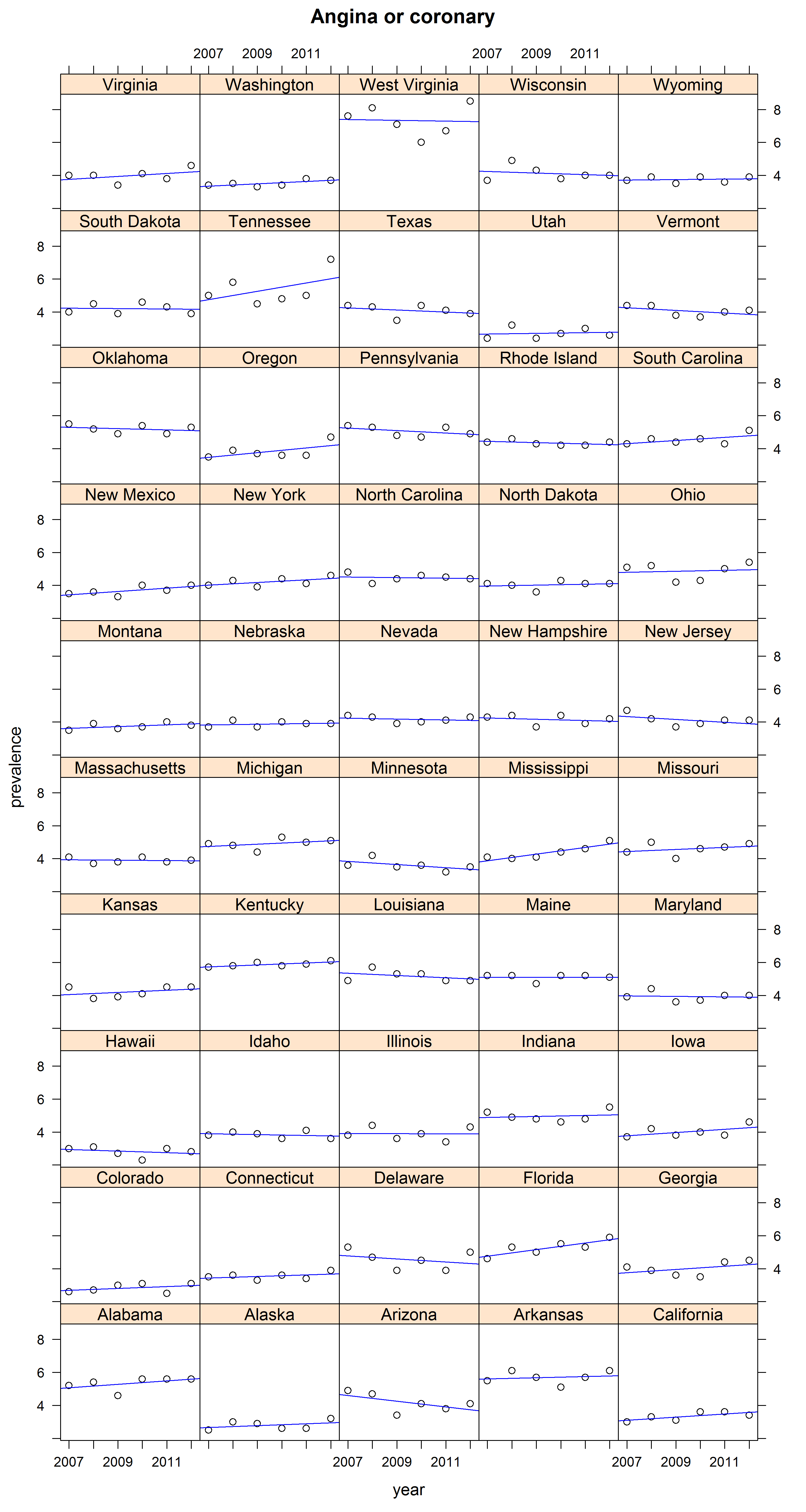


Figure A3 BRFSS reported prevalence of diseases and risk factors for year 2007-2012: Angina or Coronary Heart Disease.


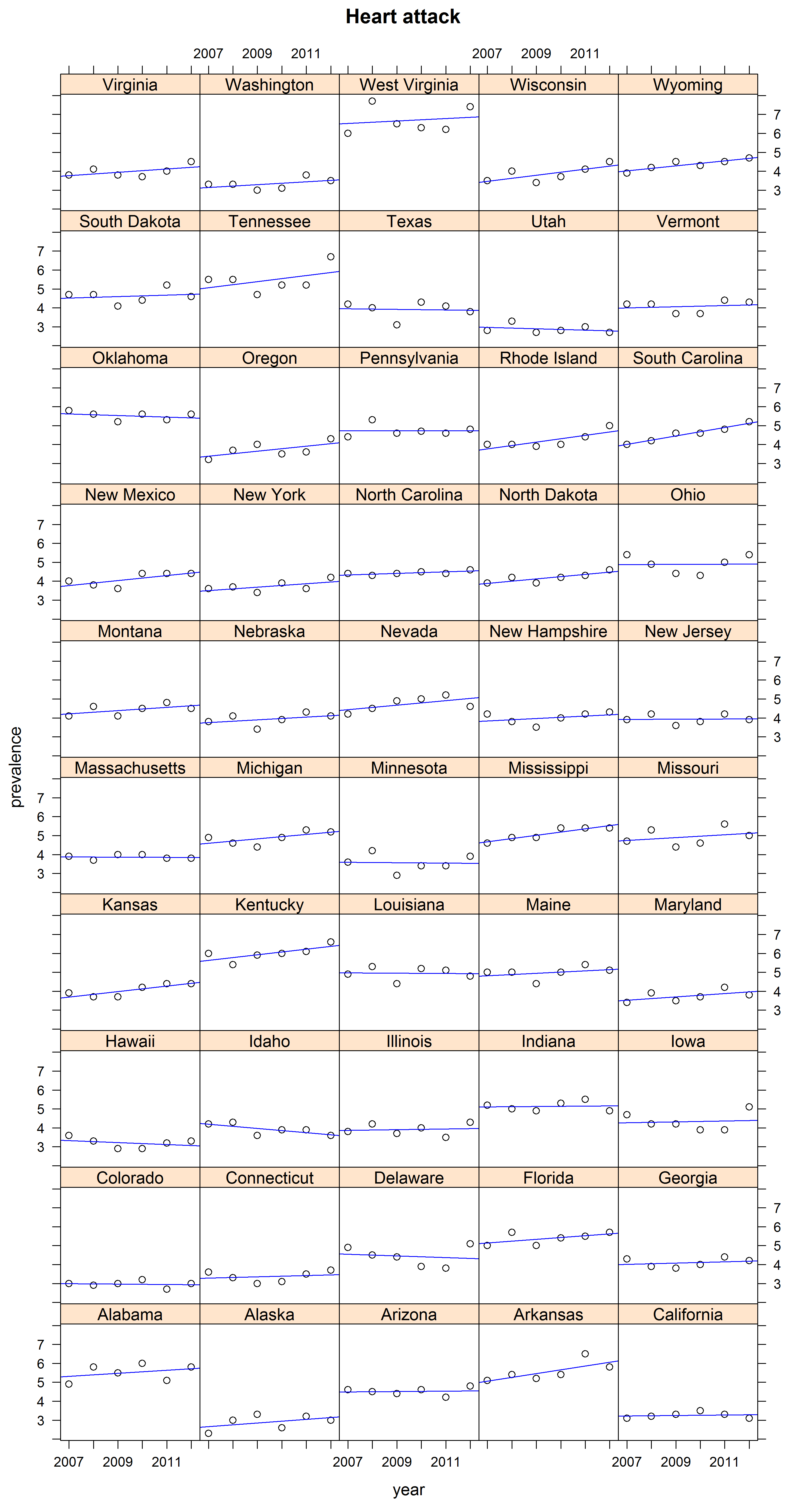


Figure A4 BRFSS reported prevalence of diseases and risk factors for year 2007-2012: Heart Attack.


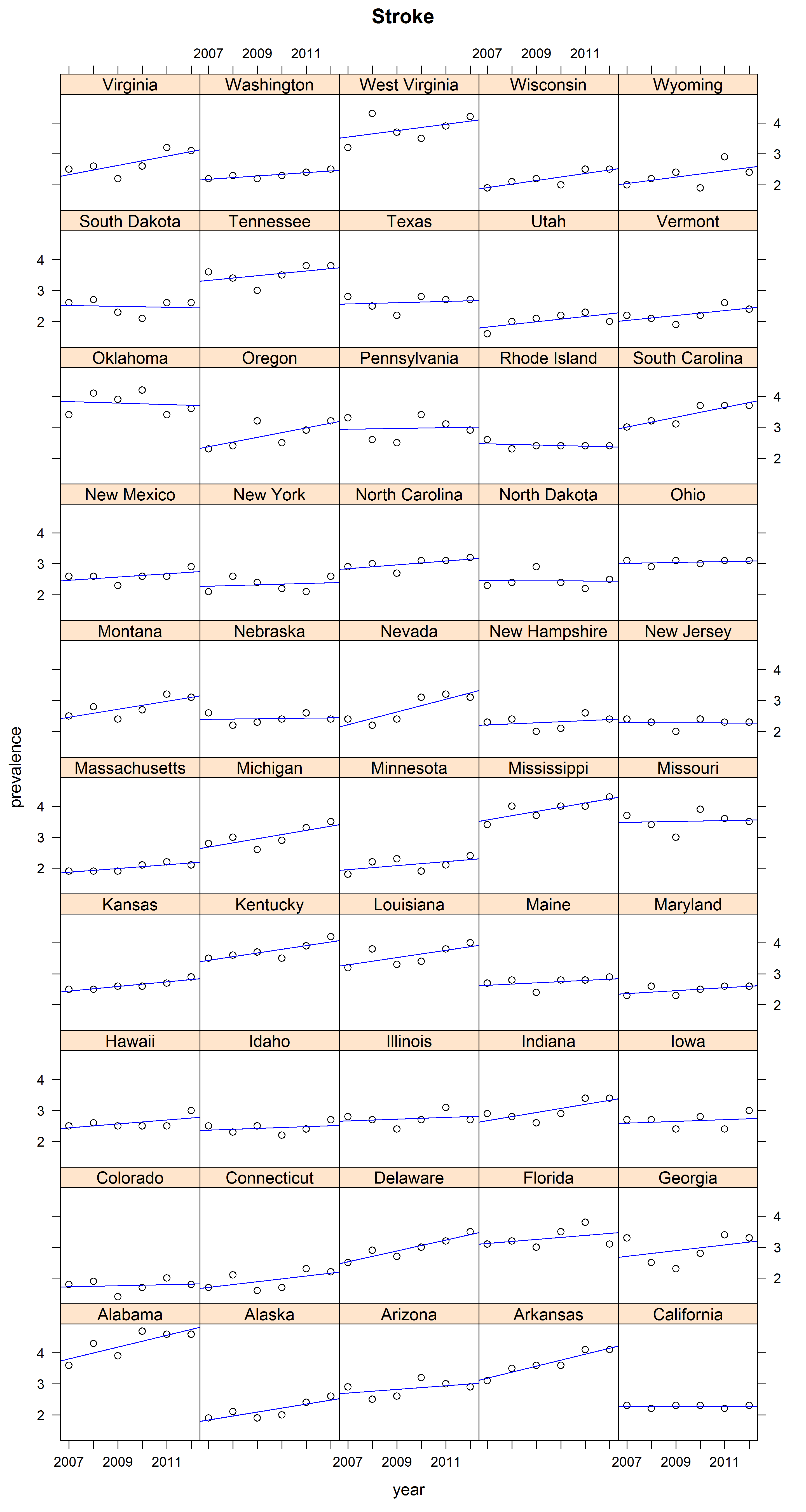


Figure A5 BRFSS reported prevalence of diseases and risk factors for year 2007-2012: Stroke.


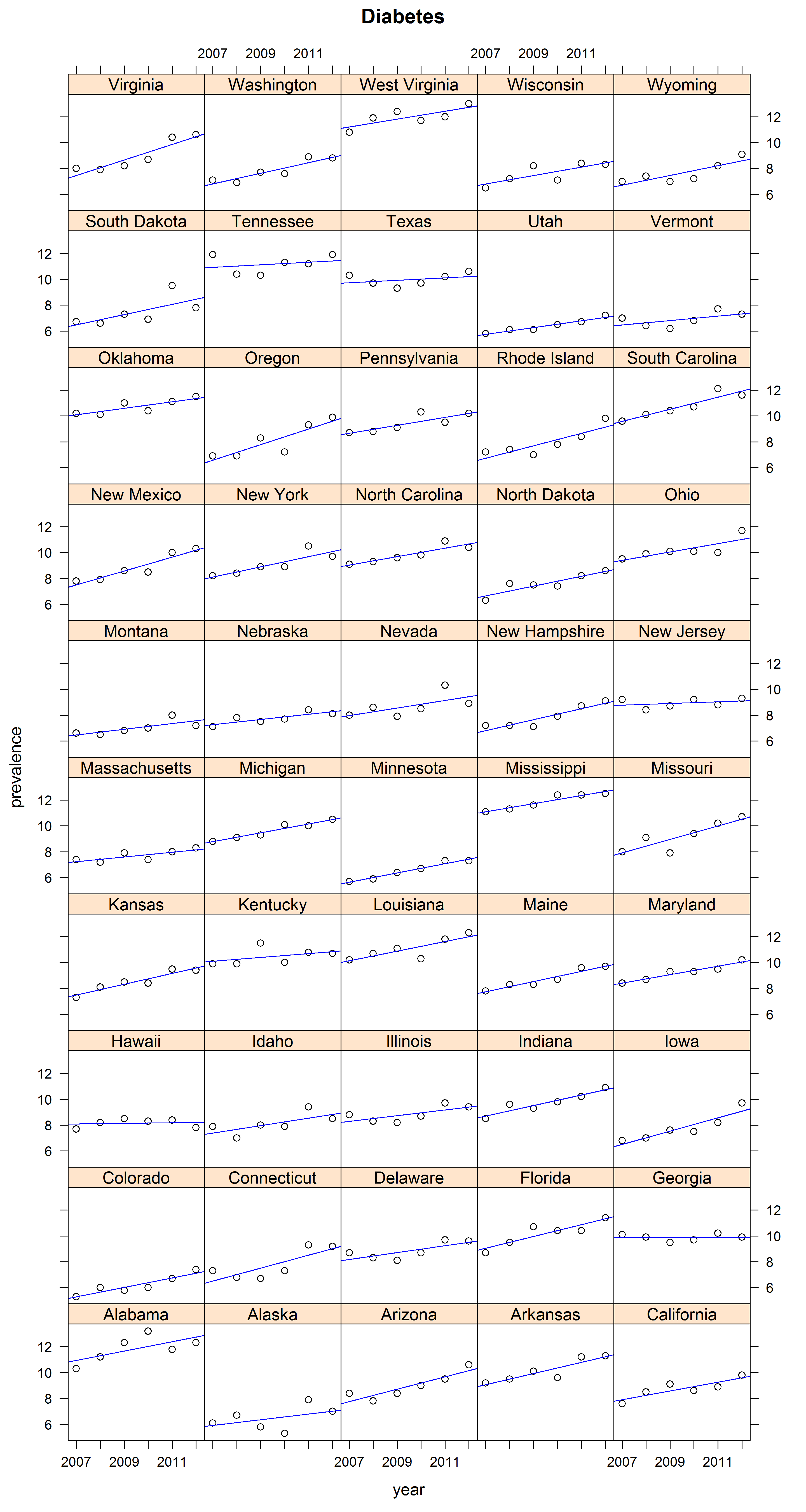


Figure A6 BRFSS reported prevalence of diseases and risk factors for year 2007-2012: Diabetes.
